# Validity of Session Rating Perceived Exertion Method for Quantifying Internal Training Load during High-Intensity Functional Training

**DOI:** 10.3390/sports6030068

**Published:** 2018-07-23

**Authors:** Ramires Alsamir Tibana, Nuno Manuel Frade de Sousa, Gabriel Veloso Cunha, Jonato Prestes, Carlos Fett, Tim J. Gabbett, Fabrício Azevedo Voltarelli

**Affiliations:** 1 Department of Physical Education, Universidade Federal de Mato Grosso (UFMT), Cuiabá 87000-000, Brazil; fettcarlos@gmail.com (C.F.); faunesp8@yahoo.com.br (F.A.V.); 2 Graduation Program on Physical Education, Catholic University of Brasilia, Brasilia 72000-000, Brazil; jonatop@gmail.com; 3 Laboratory of Exercise Physiology, Faculty Estacio of Vitoria, Vitoria 29000-000, Brazil; nunosfrade@gmail.com; 4 Medical Graduation Program, Catholic University of Brasilia, Brasilia 72000-000, Brazil; gabrielvelosoc@gmail.com; 5 Gabbett Performance Solutions, Brisbane 4011, Australia; tim@gabbettperformance.com.au; 6 Institute for Resilient Regions, University of Southern Queensland, Ipswich 4035, Australia

**Keywords:** extreme conditioning program, crossfit, high-intensity short interval, load control

## Abstract

The aim of this study was to validate the quantification of internal training load (session rating perceived exertion, sRPE) and the effect of recall timing of sRPE during high-intensity functional training (HIFT) sessions. Thirteen male HIFT practitioners (age 27.2 ± 33 years, height 177.1 ± 4.0 cm, body mass 81.1 ± 9.0 kg) were monitored during two common HIFT training sessions: Fight Gone Bad (FGB) and Fran. The Edwards summated heart-rate-zone method was used as a reference measure of internal training load. The session-RPE rating was obtained using the CR-10 scale modified by Foster. The training load calculated by the Edwards-TRIMP index was significantly higher (*p* < 0.05) during the FGB (77.7 ± 4.9) than the Fran (19.8 ± 8.4) workout. There was a strong correlation (*p* < 0.05) between the Edwards-TRIMP index and the training load calculated by the sRPE in all time frames (0, 10, 20, and 30 min post-exercise). The RPE and sRPE measured at 30 min post-exercise time frame was significant lower than 0, 10, and 20 min post-exercise for both workouts. The session-RPE method is an easy and valid tool to evaluate internal training load for high intensity functional training practitioners.

## 1. Introduction 

High-intensity functional training (HIFT) including CrossFit^®^ modality comprises a mix of elevated intensity functional movements by using basic Olympic weightlifting techniques, power training, exercises with body-weight, and aerobic training [1,2]. While HIFT are growing in the number of practitioners and popularity, debate on the safety and benefits of HIFT has emerged between the scientific literature and anecdotal reports from athletes, coaches, and physicians [3].

Recently, Tibana et al. [2] showed that two consecutive days of HIFT induced a high metabolic demand and elicited significant disturbance in pro/anti-inflammatory cytokine balance during the recovery period. The Consortium for Health and Military Performance (CHAMP) and the American College of Sports Medicine (ACSM) reported HIFT as an exercise training modality with elevated risk of injury [4]. Among possible solutions for this injury risk, the monitoring of training load should be potentially considered [4]. Although HIFT has been widely practiced by people, there is currently limited evidence of training load monitoring in athletes performing these activities [5]. 

Athletes have their internal training (ITL) load constantly evaluated to guarantee high performance. The monitoring of ITL is used to evaluate training effects during each phase of a periodization [6]. The measures of metabolic, cardiovascular, and respiratory variables are commonly applied to quantify the magnitude of ITL, while they are not practical in a “real-world” setup [7]. On the other hand, the use of session rating of perceived exertion (session-RPE) to evaluate [8] and quantify ITL is considered a potential tool in different sports [9]. The modified version of Borg’s CR-10 scale of perceived exertion can be applied for measuring training doses 30 min after bouts. The reason for evaluating RPE 30 min following exercise is to avoid light or heavy feelings from exercise sessions immediately after the termination of the trials [8]. However, some studies [10,11] revealed have that the session-RPE measured earlier than 30-min following exercise may be very similar to that obtained using the classical 30-min post exercise measurement period. This issue should be observed with caution, because 30 min may be a long period to assess session-RPE, which would be a practical limitation, as it increases time demand of HIFT practitioners [11].

Currently, there is no study that evaluated the best method for quantifying training load during HIFT. Thus, the first aim of the present study was to validate the session-RPE as a method of monitoring ITL during HIFT. We hypothesized that significant correlations would emerge between session-RPE and Edwards’ heart-rate-based methods. The second aim was to determine the effects of different time recall of session-RPE (immediately after, 10, 20, and 30 min post-exercise) to identify ITL.

## 2. Material and Methods

### 2.1. Participants

Thirteen subjects aged 27.1 ± 4.1 years with experience in HIFT were recruited by announcements about the research. All the characteristics of the subjects are shown in Table 1. Participants should not present any injuries or illness, and should not be using substances to enhance performance, and had a minimum experience of six months with HIFT. Subjects were also interviewed by researchers and revealed previous experience in resistance and aerobic training before engaging in HFT. They were advised to refrain from ingesting caffeine and alcohol for 24 h before all tests, avoid any exercise in the 48 h before the experimental sessions, and to maintain their normal daily diet during the study. All subjects signed an informed consent document and the study was approved by the University Research Ethics Committee for Human Use (2.698.225/Universidade Estácio de Sá/ UNESA/RJ) and conformed to the Helsinki Declaration on the use of human participants for research. 

### 2.2. Quantifying Training Load 

Session Rating of Perceived Exertion. Training load was calculated using the session-RPE method proposed by Foster, et al. [8] and involved multiplying the total duration of a bout or exercise session in minutes by the training intensity. Intensity was measured by a modified version of Borg’s CR-10 scale of perceived exertion, referred to session-RPE. The session-RPE score was obtained from the athletes immediately after the protocol of training and 10, 20, and 30 min after each protocol. This was in response to the question “how hard was your workout?” Training load was expressed as a single value in arbitrary units (AU).

Training impulse (TRIMP). Each subject was also provided with a Polar H10 HR-monitor (Polar Electro Oy, Finland) to measure exercise intensity during sessions. Resting heart rate (HR) was determined by instructing subjects to rest sitting on the floor for five minutes, the lowest heart rate observed during this time was deemed resting HR. After the protocols, the HR data was downloaded from the transmitters onto a computer using the Polar Flow Software (Polar Electro Oy, Finland) and then exported into Microsoft Excel (Microsoft Office 2007, Microsoft Corporation, Washington DC, USA) to calculate the training loads Edward’s TRIMP [12]. Edwards’ training load determines the internal load by measuring a product of the accumulated training duration (minute) of 5 HR zones by a coefficient related to each zone (50 to 60% of HRmax × 1; 60 to 70% of HRmax × 2; 70 to 80% of HRmax × 3; 80 to 90% of HRmax × 4; and 90–100% of HRmax × 5).

The Edwards TL formula is as follow: Edwards’ TL = duration in zone 1 × 1 + duration in zone 2 × 2 + duration in zone 3 × 3 + duration in zone 4 × 4 + duration in zone 5 × 5. 

### 2.3. Experimental Design

#### 2.3.1. Protocol 1 

This training is called “Fran” and is characterized by couplet barbell thrusters (a front squat to push press) and pull-ups following a 21–15–9 repetition scheme, where 21 thrusters were completed, then 21 pullups completed, 15 thrusters and 15 pull-ups, nine thrusters, and nine pull-ups completed for time. Variations of pullups, including butterfly and kipping, were encouraged. Thrusters were performed with 43.2 kg for males and 29.5 kg for females. The time to complete all repetitions was recorded.

#### 2.3.2. Protocol 2 

The “Fight Gone Bad” (FGB) comprises three rounds of Wall-ball (9 kg), Sumo deadlift high-pull (34 kg) Box Jump (50 cm), Push-press (34 kg), and Row (Calories). In this training session, subjects moved from each of five stations after a minute. The clock did not reset or stop between exercises. This is a five-minute round from which a one-minute break was allowed before repeating. On call of “rotate,” the athletes moved immediately to the next station. The best score (number of repetitions) was recorded.

#### 2.3.3. Lactate Analysis 

Lactate (LAC) was determined by photometric reflectance on a validated Portable Accutrend Plus system (Roche, Sao Paulo, Brazil). Capillary blood samples were collected through transcutaneous puncture on the medial side of the tip of the middle finger using a disposable hypodermic lancet. Blood LAC concentrations were measured before and immediately after each protocol of exercise. 

#### 2.3.4. Statistical Analyses

The data are expressed as means ± standard deviation (SD). The Shapiro-Wilk test was applied to check for normality distribution of the variables assessed. RPE and session-RPE did not meet the assumption of normal distribution and in this case non-parametric tests were used. Paired sample *t*-test was used to compare TRIMP Edwards index, heart rate and blood lactate concentration between workouts. Wilcoxon test was used to compare RPE and session-RPE between workouts. Non-parametric repeated measures were used to compare RPE and session-RPE between post-exercise periods following workouts. Correlation between TRIMP Edwards index and session-RPE at different time points were evaluated using Spearman product moment coefficients. Cohen’s effect size statistic was used for assigning strength of association between the variables (to evaluate the power of the association) and to evaluate the effect size of the differences between workouts. The power of the sample size was determined using G*Power version 3.1.3, based on the correlation between TRIMP Edwards index and session-RPE. Considering the sample size of this study and an alpha error of 0.05, the lowest power (1 − β) achieved in this correlation was 0.99. The level of significance was *p* ≤ 0.05 and SPSS version 20.0 (Somers, NY, USA) software was used.

## 3. Results

Table 1 presents the anthropometric and performance measurements of the subjects. Immediately after the 2 exercise bouts, HR and blood lactate concentration did not present a statistically significant difference (*p* ≥ 0.05), although the time of exercise bouts were very different (approximately 4 min for Fran and 17 min for FGB; Table 2). 

**Figure 1:**
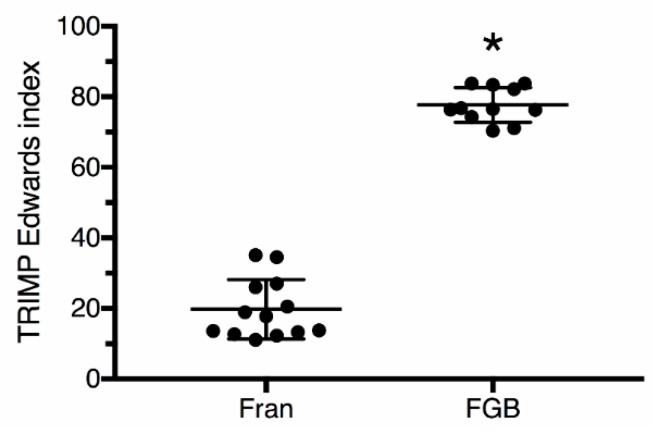
Training load calculated by the TRIMP Edwards index during Fran and FGB workouts. * *p* < 0.05 for Fran; Cohen’s *d* = 6.90.

**Figure 2:**
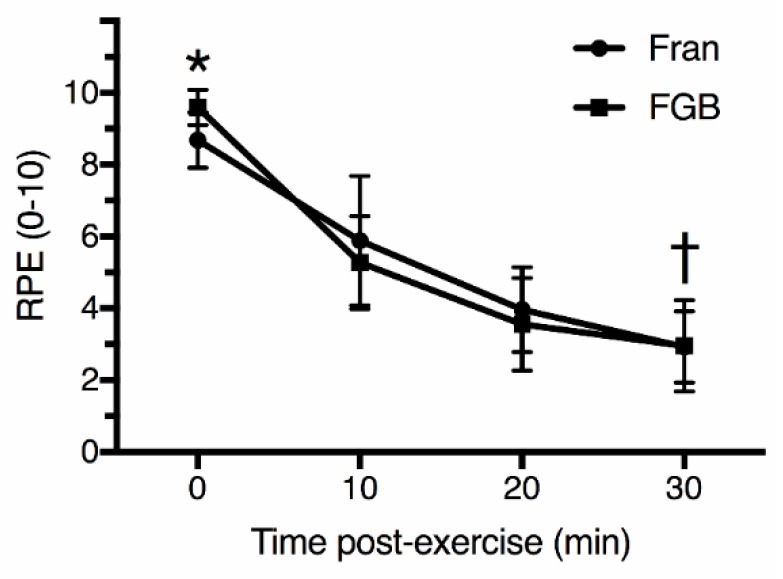
Rating of perceived exertion (RPE) at different post-exercise times after Fran and FGB workouts. * *p* < 0.05 between workouts; † *p* < 0.05 for 0, 10 and 20 min post-exercise for both workouts. 0 min Cohen’s *d* = 1.15; 10 min Cohen’s *d* = 0.34; 20 min Cohen’s *d* = 0.35; 30 min Cohen’s *d* = 0.03.

**Figure 3:**
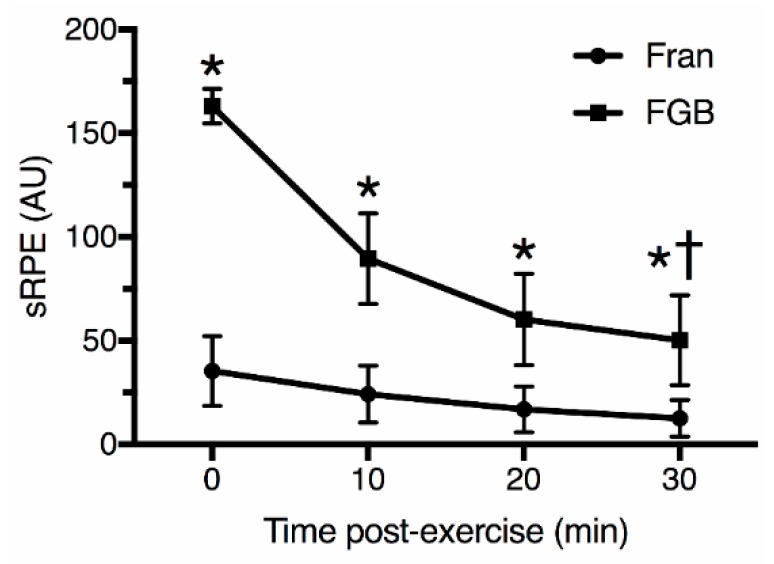
Session rating of perceived exertion (sRPE) at different post-exercise times after Fran and FGB workouts. * *p* < 0.05 between workouts; † *p* < 0.05 for 0, 10 and 20 min post-exercise for both workouts. 0 min Cohen’s *d* = 7.59; 10 min Cohen’s *d* = 4.76; 20 min Cohen’s *d* = 3.95; 30 min Cohen’s *d* = 4.29.

The training load, calculated by the TRIMP index, was significantly higher (*p* < 0.05) during the FGB (77.7 ± 4.9) than the Fran (19.8 ± 8.4) protocols (Figure 1). There was a strong correlation (*p* < 0.05) between the TRIMP index and the training load calculated by the session-RPE in all time frames (0, 10, 20 and 30 min post-exercise; Table 3). However, there was significant differences (*p* < 0.05) in RPE (Figure 2) and training load (Figure 3), calculated by the session-RPE, related to post-exercise rating times for the two protocols. The RPE and session-RPE measured at the classical 30 min post-exercise time frame was significantly lower than 0, 10, and 20 min post-exercise for both protocols. When the workouts were compared, the RPE immediately after the FGB (9.6 ± 0.5) was significantly higher than the Fran (8.7 ± 0.8) protocol. No other significant differences (*p* ≥ 0.05) were observed between workouts for RPE. The session-RPE during all time frames after the FGB was significantly higher (*p* < 0.05) than the Fran workout.

## 4. Discussion

The main findings of this study were that the session-RPE method is an easy and valid tool to evaluate ITL in high intensity functional training practitioners, allowing coaches to efficiently monitor their training plans. Moreover, the classical 30-min post-exercise time-point should be used to prevent high ITL load measures soon after HFT sessions, possibly due to the high metabolic demand imposed by this type of exercise.

We are not aware of previous studies evaluating the method of the session-RPE as a simple and real-world tool to quantity ITL through examining the similarity between the session-RPE and Edward’s TRIMP during different HIFT sessions. Our findings are in agreement with other investigations that showed the validity of this method in several sport disciplines including Judo, boxing, rugby, tennis, and volleyball [9]. Previously, two studies had used this method to quantify the ITL in CrossFit practitioners [13,14]. Tibana, et al. [13] showed in a case study that session-RPE was able to distinguish different ITL at times of tapering, overloading and recovery during an 11-week training program. Moreover, Williams, et al. [14] investigated the ITL (heart rate variability and session-RPE) and risk of overreaching issues in CrossFit athletes across a 16-week period. The results reported by the authors showed that the risk of overreaching issues was elevated when a “low” natural logarithm of the square root of the mean sum of the squared differences between R–R intervals (Ln rMSSD week) was seen in combination with a “high” acute-to-chronic workload ratio, measured by the session-RPE.

Interestingly, it has been demonstrated that an HIFT session resulted in increased acute oxidative stress [15], metabolic and inflammatory stress [2], cardiovascular and perceived exertion response [13], sympathetic nervous system markers (i.e., plasma Epinephrine and Norepinephrine), and depression in Ln rMSSD [16]. Due to exacerbated physiological responses, the Consortium for Health and Military Performance (CHAMP) and the ACSM considered HIFT as an exercise training modality with increased risk of injury and suggested, the monitoring of training load to reduce injury risk [4]. The results of the present study may be useful for coaches to systematically monitor practitioners internal training load to improve performance, and decrease excessive training syndrome, injuries, and illness during a training period [17]. 

Regarding the recall of intensity at different times post-session, in the present study the RPE and session-RPE measured at the classical 30 min post-exercise time frame was significant lower than 0, 10, and 20 min post-exercise for both workouts. The present results are in conflict with those of Christen et al. [10] and Uchida et al. [11], who demonstrated that the session-RPE obtained earlier than 30-min after the exercise was similar to that obtained using the classical 30-min post exercise measurement period. However, the studies of Christen et al. [10] and Uchida et al. [11] analyzed the ITL during cycle ergometer and boxing, respectively. These sports may result in different ITL as compared to highly fatiguing HIFT sessions. Singh, Foster, Tod, and McGuigan [18] evaluated the efficacy of applying the session-RPE to determine physical effort in trained males submitted to distinct types of resistance-training. The authors showed that the session-RPE is a beneficial way to evaluate the intensity resistance-training sessions. Furthermore, similarly to the present study, there was a significant difference between the session-RPE values at different time intervals (recorded at 10-min intervals until 30 min post-exercise) and session-RPE values at the 30-min time-point. Thus, for HIFT activities, session-RPE should be evaluated 30 min after the termination of a training session to prevent particularly hard or light elements following the trial from distorting the entire rating of the session.

Despite the interesting findings of this study, some limitations need to be mentioned. First, the reduced number of HIFT sessions. Second, the time recall of session-RPE was limited until 30 min after exercise. Third, it should be noted, that these results should be considered only for trained men subjects. Therefore, our findings may not be directly transferable to untrained or to females. Finally, we only assessed metabolic conditioning sessions of HIFT. 

## 5. Conclusions

In conclusion, session-RPE is an effective form to determine ITL during HIFT session and should be used by coaches to improve individual training prescription and to avoid excessive training loads, which could lead to overtraining. Moreover, the 30-min time-point should be used to prevent high ITL loads measures soon after HIFT sessions, possibly due to the high metabolic demand imposed by this type of exercise. If coaches or practitioners choose to use time-points earlier than 30 min, they should try to repeat the measure at the same period to avoid different ITL values. This may also be dependent on the daily routine. 

## Figures and Tables

**Table 1:** Anthropometric and performance measurements of the subjects (mean ± SD).

Characteristic	(n = 13)
Anthropometric variables	-
Age, years	27.2 ± 33
Height, cm	177.1 ± 4.0
Mass, kg	81.1 ± 9.0
BMI, kg/m^2^	25.9 ± 3.0
Body fat, %	11.3 ± 4.7
Performance variables	-
VO_2_max, mL·(kg·min)^−1^	51.8 ± 3.3
Back squat, kg	149.1 ± 30.9
Front squat, kg	131.9 ± 28.8
Clean, kg	114.8 ± 21.6
Snatch, kg	96.6 ± 16.5

**Table 2:** Performance results, heart rate and blood lactate concentration after Fran and Fight Gone Bad (FGB) workouts (mean ± SD).

	Fran	FGB
Time, s	4.06 ± 1.99	-
Repetitions	-	291.5 ± 34.8
Heart rate, bpm		
Before	68.8 ± 8.8	64.9 ± 14.1
Immediately after	182.0 ± 5.2 *	184.4 ± 4.1 *
Blood lactate concentration, mmol·L^−1^		
Before	2.3 ± 0.6	2.2 ± 0.8
Immediately after	17.8 ± 4.9 *	17.2 ± 3.5 *

* *p* ≤ 0.05 for Fran workout.

**Table 3:** Spearman product moment correlation between Training impulse (TRIMP) Edwards and session rating of perceived exertion (sRPE) at different time frames post-exercise.

	TRIMP Edwards		
	r	*p*-Value	Power (1 − β)
sRPE 0 min post-exercise	0.865	<0.0005	1.00
sRPE 10 min post-exercie	0.836	<0.0005	0.99
sRPE 20 min post-exercise	0.858	<0.0005	1.00
sRPE 30 min post-exercise	0.834	<0.0005	0.99
